# DISPARITIES IN HEALTH STATUS, HEALTH BEHAVIORS AND PREVENTIVE HEALTH SERVICES OF OLDER ADULTS WITH DIABETES

**DOI:** 10.1093/geroni/igz038.3503

**Published:** 2019-11-08

**Authors:** Sang Ah Chun

**Affiliations:** University at Albany, SUNY, Albany, New York, United States

## Abstract

The prevalence of diabetes among older adults has increased substantially and health complications resulting from diabetes have significant adverse effects on health status of older adults. While diabetes cannot be cured, it can be managed successfully with healthy lifestyle choices. The purpose of this study is to examine the disparities in health status, health behaviors, and preventive health services for older adults with diabetes. This study used data from the 2018 Behavioral Risk Factor Surveillance System. The sample included older adults 50 and over. Health behaviors included exercise, smoking, and heavy alcohol drinking. Preventive health services included dental visit, flu shot, and colorectal cancer screening. Chi-Square analysis and weighted multivariate logistic regression was performed. Not surprisingly, older adults with diabetes were significantly more likely to be in poor health than those without diabetes. Compared to non-diabetic group, older adults with diabetes were more likely to have had no exercise in the previous month. Interestingly, more older adults with diabetes reported having visited dentist, had flu shot and colonoscopy than those without diabetes. In both groups, older adults who presented health behaviors and received preventive health services were more likely to report good health compared to those who did not. The results suggest that further efforts are needed to address the health disparities for older adults with diabetes. Given the risk of comorbidities and its complications for older adults with diabetes, further research should be directed toward designing better health promotion programs and policies for older adults with diabetes.

